# Evaluation of a new dynamic real-time visualization 25 kHz swept-source optical coherence tomography based biometer

**DOI:** 10.1186/s40662-024-00377-2

**Published:** 2024-03-04

**Authors:** Jinjin Yu, Xuanqiao Lin, Xiaomin Huang, Zhenyu Xu, Rui Ning, Kexin Li, Giacomo Savini, Domenico Schiano-Lomoriello, Xingtao Zhou, Jinhai Huang

**Affiliations:** 1 Eye Institute and Department of Ophthalmology, Eye & ENT Hospital, Fudan University, N No. 19 Baoqing Road, Xuhui District, Shanghai, 200031 China; 2 Shanghai Research Center of Ophthalmology and Optometry, Shanghai, China; 3 Ningbo No. 2 Hospital, Ningbo, Zhejiang China; 4 Eye Hospital and School of Ophthalmology and Optometry, Wenzhou Medical University, Wenzhou, Zhejiang China; 5 IRCCS Bietti Foundation, Rome, Italy; 6 NHC Key Laboratory of Myopia (Fudan University), Key Laboratory of Myopia, Chinese Academy of Medical Sciences, Shanghai, China

**Keywords:** Swept-source optical coherence tomography, Ocular biometric, Repeatability, Reproducibility, Agreement

## Abstract

**Background:**

To evaluate the intraobserver repeatability and interobserver reproducibility of a newly developed dynamic real-time visualization 25 kHz swept-source optical coherence tomography (SS-OCT) based biometer (ZW-30, TowardPi Medical Technology Ltd, China) and compare its agreement with another SS-OCT based biometer (IOLMaster 700, Carl Zeiss Meditec AG, Jena, Germany).

**Methods:**

Eighty-two healthy right eyes were enrolled in this prospective observational study. Measurements were repeated for three times using the ZW-30 and IOLMaster 700 in a random order. Obtained parameters included axial length (AL), central corneal thickness (CCT), aqueous depth (AQD), anterior chamber depth (ACD), lens thickness (LT), mean keratometry (Km), astigmatism magnitude (AST), vector J_0_, vector J_45_, and corneal diameter (CD). The within-subject standard deviation (Sw), test–retest (TRT) variability, coefficient of variation (CoV), and intraclass correlation coefficient (ICC) were adopted to assess the intraobserver repeatability and interobserver reproducibility. The double-angle plot was also used to display the distribution of AST. To estimate agreement, Bland–Altman plots were used.

**Results:**

For the intraobserver repeatability and interobserver reproducibility, the Sw, TRT and CoV for all parameters were low. Meanwhile, the ICC values were all close to 1.000, except for the J_45_ (ICC = 0.887 for the intraobserver repeatability). The double-angle plot showed that the distribution of AST measured by these two devices was similar. For agreement, the Bland–Altman plots showed narrow 95% limits of agreements (LoAs) for AL, CCT, AQD, ACD, LT, Km AST, J_0_, J_45_, and CD (− 0.02 mm to 0.02 mm, − 7.49 μm to 8.08 μm, − 0.07 mm to 0.04 mm, − 0.07 mm to 0.04 mm, − 0.07 mm to 0.08 mm, − 0.16 D to 0.30 D, − 0.30 D to 0.29 D, − 0.16 D to 0.16 D, − 0.23 D to 0.13 D, and − 0.39 mm to 0.10 mm, respectively).

**Conclusions:**

The newly dynamic real-time visualization biometer exhibited excellent intraobserver repeatability and interobserver reproducibility. The two devices both based on the SS-OCT principle had similar ocular parameters measurement values and can be interchanged in clinical practice.

## Background

Regarding the diagnosis and treatment of any eye disease, such as the calculation of intraocular lens (IOL) power in cataract surgery [1], preoperative evaluation and surgical plan design of refractive surgery [2], and phakic IOLs sizing [3], accurate measurements of eye parameters are required. To this end, biometric technologies have been developed continually and widely used in clinical setting.

Currently, swept-source optical coherence tomography (SS-OCT) is the latest piece of technology. There are several commercially available SS-OCT optical biometers that work at long wavelengths with high penetration (1035 nm to 1310 nm), producing long-range OCT images starting from the cornea to the posterior lens/retina. The IOLMaster 700 (Carl Zeiss Meditec AG, Jena, Germany) was the first SS-OCT based optical biometer. Its measurement repeatability and agreement with other instruments, including a partial coherence interferometry (PCI) based optical biometer (IOLMaster 500, Carl Zeiss Meditec AG, Jena, Germany) [4], an optical low-coherence reflectometry (OLCR) based optical biometer (Lenstar LS900, Haag-Streit, Köniz, Switzerland) [5], and an optical low-coherence interferometry (OLCI) based optical biometer (Aladdin, Topcon, Tokyo, Japan) [6] have been confirmed. By providing a two-dimensional (2D) image of a small central macular area, it helps to determine if the patient has good fixation during data capture.

The ZW-30 (TowardPi Medical Technology Ltd, China) is a newly developed device that combines SS-OCT with a tunable laser wavelength centered on 1060 nm (the bandwidth is greater than 40 nm), scanning at a speed of 25,000 times/s. Unlike the IOLMaster 700 which is unable to capture the entire length of the eye during preview, this new device allows for real-time and dynamic viewing of the entire eye’s axis during measurement and gives more accurate measurements.

Since its recent release, the reliability of the device has yet to be evaluated in detail before its use in clinical practice. This study aims to first investigate the repeatability and reproducibility of ocular parameters measurement obtained by this new SS-OCT based device, and then compare its agreement with the IOLMaster 700.

## Methods

### Study population

This prospective observational study enrolled patients who underwent myopia refractive surgery at the Eye & ENT Hospital of Fudan University. The study protocol was approved by the Ethics Committee of the Eye & ENT Hospital of Fudan University (No. 2021175) and in accordance with the tenets of the Declaration of Helsinki. Each patient signed the informed consent after understanding the content of this research.

All patients received complete ophthalmic examinations, including corrected distance visual acuity (CDVA), slit-lamp examination, non-contact tonometry and direct fundus examination without mydriasis. The inclusion criteria were as follows: (1) age older than 18 years and CDVA ≥ 20/20, (2) no pathological changes in the anterior segment (such as corneal haze, keratoconus, and cataract), (3) no posterior segment diseases (such as vitreous hemorrhage, retinal detachment, and optic neuropathy), (4) no ocular surgery, and (5) no systematic disease that may affect the eye. Patients who could not maintain appropriate eye fixation during the data acquisition and those who stopped wearing soft contact lenses less/rigid contact lenses for a period shorter than 2 weeks/4 weeks were excluded.

### The SS-OCT devices

#### IOLMaster 700

The IOLMaster 700 is a SS-OCT based optical biometer launched in 2014; it uses the light of the central wavelength of 1,050 nm (varying from 1,035 nm to 1,095 nm) with a 44 mm scan depth. Six images are captured from six orientations (0°, 30°, 60°, 90°, 120°, and 150°) for measurements of axial parameters including axial length (AL), corneal central thickness (CCT), anterior chamber depth (ACD), and lens thickness (LT). The keratometric readings are calculated by a telecentric technique, which projects the 950 nm light source onto the cornea and analyzes 18 reference points at three zones (1.5 mm, 2.5 mm, and 3.5 mm optical zones). The device uses an 800 nm light-emitting diode (LED) source to obtain the horizontal pupil diameter (PD) and corneal diameter (CD, the diameter of the visible corneal area from limbus to limbus) distance. From the whole-eye B-scan images, we can visualize a small central 1.0 mm zone macular scan.

#### ZW-30

**Fig. 1:**
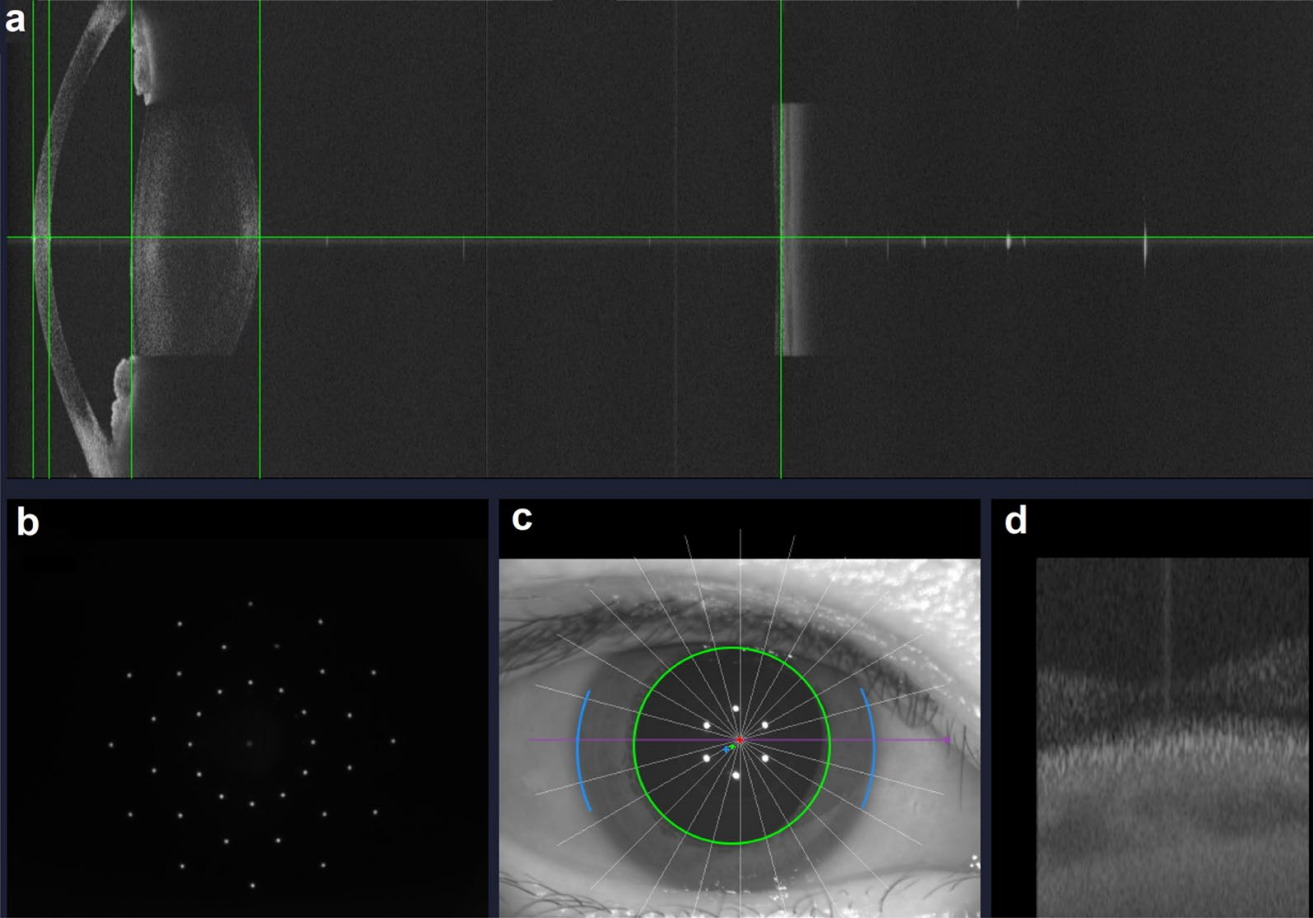
The shooting interface of the ZW-30. **a** The complete longitudinal section of the eye; **b** The shooting interface of corneal curvature; **c** The shooting interface of corneal diameter and pupil diameter; **d** The visualization of the macular zone

**Fig. 2:**
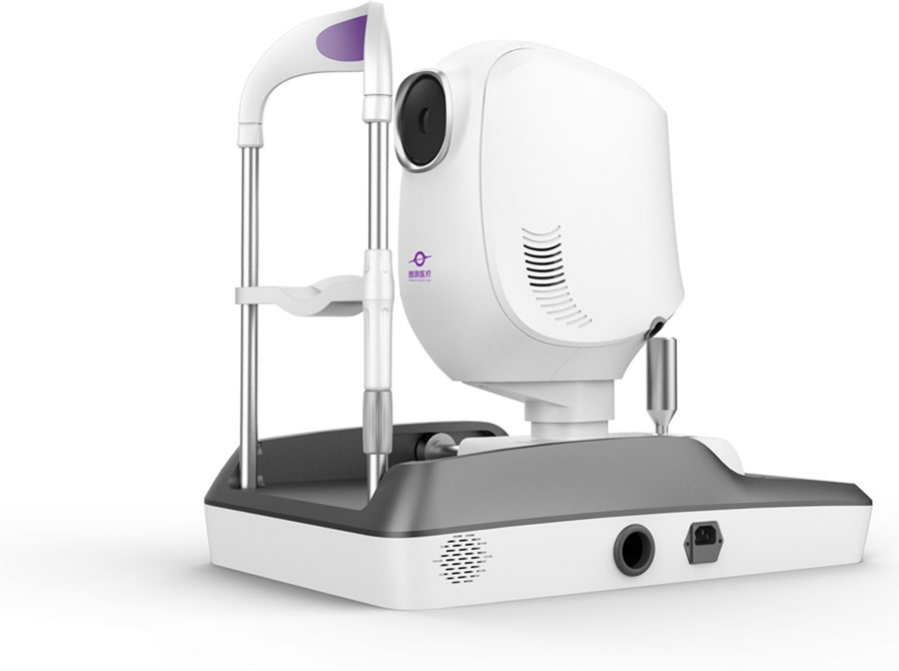
Appearance of the ZW-30

The ZW-30 device is a new optical biometer based on SS-OCT technology with a 1060 nm central wavelength of light. Its dynamic real-time visualization full-eye OCT scanning allows a largest axial length measurement scope (range from 14 mm to 45 mm) as well as an extremely large lateral scanning length (12 mm) and visualization of the macular zone. The measurement is quick (less than 0.5 s) with a scanning rate of 25,000 A-scans/s. The device measures the AL, ACD, CCT, LT, and vitreous chamber depth using SS-OCT technology in 12 scan lines at 0°, 15°, 30°, 45°, 60°, 75°, 90°, 105°, 120°, 135°, 150° and 165°. AL measurements are the average values of 16 scans in each of 12 meridians. AL is measured using two indexes: a specific refractive index for each segment of the eye (cornea = 1.376; aqueous = 1.336; lens = 1.413; vitreous = 1.336) and an equivalent refractive index. In the current study, the latter is used. The corneal curvature is acquired through a multidot keratometer by 36 reflected spots at the 1.5 mm, 2.5 mm, and 3.5 mm central zone projected on the corneal surface from the inner circle to the outer circle, which is designed with three concentric near-infrared LED lights with a central wavelength of 850 nm and 12 lights per circle. Three single measurements are taken, and the final average keratometry readings are calculated (2.5 mm). The device uses a 750 nm illumination LED source to obtain the horizontal PD and CD distance. Figures 1 and 2 show the shooting interface and the appearance of the device, respectively.

### Measurement procedure

All subjects received biometric measurements by one experienced operator (JY) using the ZW-30 and IOLMaster 700 in a random order. Each eye was measured three times consecutively. Later, another experienced operator (XL) measured subjects three times using ZW-30 as well. Before measuring, calibration was conducted for both instruments; then, the subjects had to place their chin on the chin rest, place their forehead against the foreheadsupport and look at the fixation point. When the measurement was about to begin, the subjects were told to blink their eyes to allow a uniform coating of tear film. The testing environment was in a dim room with the testing performed within 10 min. To avoid the impact of correlation between the two eyes on the results, we only selected the right eye for analysis [7]. Parameters measured in this study were AL, CCT, aqueous depth (AQD, the distance from corneal endothelium to lens epithelium), ACD (the distance from corneal epithelium to lens epithelium), flattest keratometry (Kf), steepest keratometry (Ks), and CD. Mean keratometry (Km) was calculated as the mean value of the Kf and Ks; corneal astigmatism magnitude (AST) was calculated as the difference between Ks and Kf. Corneal AST was further analyzed in vector analysis (J_0_: power vectors along the 0-degree meridian; J_45_: power vectors along the 45-degree meridian) to find out the changes in cylinder power and cylinder axis [8]: J_0_ =  − (Ks − Kf)/2 × Cos2ɑ and J_45_ =  − (Ks − Kf)/2 × Sin2ɑ (where ɑ represents the cylindrical axis).

A double-angle plot was used to display the distribution of corneal AST, where the centroid is the vectoral center of the data, the 95% confidence ellipse of the dataset is the 95% confidence interval (CI) of the observations [1.96 standard deviation (SD) for a normal distribution], and the 95% confidence ellipse of the centroid is the 95% CI of the mean (1.96 SD of the mean for a normal distribution) [9].

### Statistical analysis

Data were statistically analyzed using SPSS for Windows (version 21.0, IBM corporation, USA) and Excel software 365 (Microsoft Corp., USA). The Kolmogorov–Smirnov test was used to examine the normal distribution of data, which were expressed by mean ± SD. The intraobserver repeatability and interobserver reproducibility were investigated by the within-subject standard deviation (Sw), test–retest (TRT) variability, coefficient of variation (CoV), and intraclass correlation coefficient (ICC). The TRT was obtained by multiplying the Sw by 2.77, indicating that among 95% of the subjects the difference between the two measurements is less than 2.77 Sw. The CoV is expressed as a percentage and can be obtained by dividing the Sw by the mean. The lower the CoV, the higher the reliability. The ICCs is the ratio of the between-subject variance to the sum of the pooled within-subject variance and the between-subject variance. An ICC value close to 1.000 indicates a smaller variance between repeated measurements. A paired t-test was used to compare the average values of repeated measurements of the two devices. Double-angle plots were entered in the AST double angle plot tool available on the American Society of Cataract and Refractive Surgery (ASCRS) website (https://ascrs.org/tools/astigmatism-double-angle-plot-tool) to obtain the distribution of corneal AST. Agreement between the ZW-30 and IOLMaster 700 was estimated by Bland–Altman plots and the 95% limits of agreement (LoA) (defined as the mean difference ± 1.96 SD of the differences between the paired devices). Statistical significance was set as *P* < 0.05.

## Results

The study included 82 eyes of 82 healthy subjects (37 males and 45 females) with an average age of 27.35 ± 7.00 years (range: 18 to 47 years). The mean spherical equivalent was − 5.55 ± 2.19 diopter (D) ranging from − 1.25 D to − 12.63 D.

### Intraobserver repeatability and interobserver reproducibility of the ZW-30

Tables 1 and 2 show the intraobserver repeatability and interobserver reproducibility analysis of the measurements taken by the ZW-30. The Sw, TRT and CoV for AL, CCT, ACD, AQD, LT, Km, AST, J_0_ and CD were low. Meanwhile, the ICC values were all close to 1.000 (≥ 0.966). As for the repeatability of J_45_, the ICC was relatively small, ranging from 0.887 to 0.899, while for reproducibility, the ICC value was 0.991.

**Table 1:** Intraobserver repeatability of the ZW-30

Parameter	Observer	Mean ± SD	S_w_	TRT	CoV (%)	ICC (95% CI)
AL (mm)	1st	25.76 ± 1.10	0.01	0.02	0.02	1.000 (1.000 to 1.000)
	2nd	25.79 ± 1.16	0.01	0.03	0.04	1.000 (1.000 to 1.000)
CCT (μm)	1st	543.65 ± 30.34	1.84	5.11	0.34	0.996 (0.995 to 0.998)
	2nd	537.73 ± 31.39	1.91	5.30	0.36	0.996 (0.994 to 0.998)
AQD (mm)	1st	3.10 ± 0.25	0.01	0.04	0.46	0.997 (0.995 to 0.998)
	2nd	3.10 ± 0.27	0.02	0.05	0.56	0.996 (0.993 to 0.998)
ACD (mm)	1st	3.64 ± 0.25	0.01	0.04	0.39	0.997 (0.995 to 0.998)
	2nd	3.64 ± 0.27	0.02	0.05	0.46	0.996 (0.994 to 0.998)
LT (mm)	1st	3.68 ± 0.27	0.02	0.05	0.51	0.995 (0.993 to 0.997)
	2nd	3.72 ± 0.28	0.02	0.06	0.57	0.994 (0.991 to 0.997)
Km (D)	1st	43.36 ± 1.33	0.12	0.32	0.27	0.992 (0.989 to 0.995)
	2nd	43.36 ± 1.45	0.11	0.30	0.25	0.995 (0.991 to 0.997)
AST (D)	1st	1.28 ± 0.73	0.12	0.34	–	0.972 (0.960 to 0.981)
	2nd	1.10 ± 0.66	0.12	0.34	–	0.966 (0.946 to 0.980)
J_0_ (D)	1st	− 0.59 ± 0.39	0.06	0.18	–	0.974 (0.963 to 0.982)
	2nd	− 0.48 ± 0.37	0.07	0.19	–	0.967 (0.947 to 0.981)
J_45_ (D)	1st	− 0.03 ± 0.18	0.06	0.18	–	0.887 (0.842 to 0.922)
	2nd	− 0.03 ± 0.20	0.07	0.18	–	0.899 (0.842 to 0.939)
CD (mm)	1st	11.89 ± 0.40	0.07	0.20	0.61	0.967 (0.953 to 0.978)
	2nd	11.96 ± 0.42	0.08	0.22	0.65	0.967 (0.947 to 0.981)

*AL* = axial length; *CCT* = central corneal thickness; *AQD* = aqueous depth; *ACD* = anterior chamber depth; *LT* = lens thickness; *Km* = mean keratometry; *AST* = astigmatism; *CD* = corneal diameter; *SD* = standard deviation; *S*_w_ = within-subject standard deviation; *TRT* = test–retest repeatability (2.77 S_w_); *CoV* = within-subject coefficient of variation; *ICC* = intraclass correlation coefficient; *CI* = confidence interval

**Table 2:** Interobserver reproducibility of the ZW-30

Parameter	Mean ± SD	S_w_	TRT	CoV (%)	ICC (95% CI)
AL (mm)	25.79 ± 1.16	0.00	0.01	0.02	1.000 (1.000 to 1.000)
CCT (μm)	537.73 ± 31.18	1.45	4.02	0.27	0.998 (0.996 to 0.999)
AQD (mm)	3.10 ± 0.27	0.01	0.03	0.40	0.998 (0.996 to 0.999)
ACD (mm)	3.64 ± 0.27	0.01	0.03	0.33	0.998 (0.996 to 0.999)
LT (mm)	3.72 ± 0.28	0.01	0.04	0.34	0.998 (0.996 to 0.999)
Km (D)	43.36 ± 1.46	0.08	0.23	0.19	0.997 (0.994 to 0.998)
AST (D)	1.10 ± 0.65	0.10	0.27	–	0.978 (0.961 to 0.988)
J_0_ (D)	− 0.48 ± 0.36	0.06	0.16	–	0.975 (0.955 to 0.986)
J_45_ (D)	− 0.03 ± 0.20	0.05	0.13	–	0.945 (0.903 to 0.969)
CD (mm)	11.95 ± 0.42	0.04	0.11	0.34	0.991 (0.984 to 0.995)

*AL* = axial length; *CCT* = central corneal thickness; *AQD* = aqueous depth; *ACD* = anterior chamber depth; *LT* = lens thickness; *Km* = mean keratometry; *AST* = astigmatism; *CD* = corneal diameter; *SD* = standard deviation; *S*_w_ = within-subject standard deviation; *TRT* = test–retest repeatability (2.77 S_w_); *CoV* = within-subject coefficient of variation; *ICC* = intraclass correlation coefficient; *CI* = confidence interval

### Comparison between the ZW-30 and IOLMaster 700

**Fig. 3:**
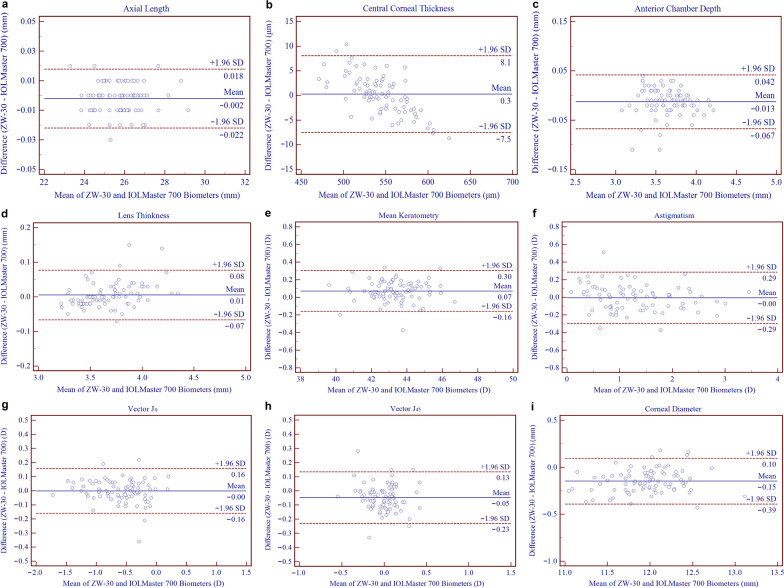
Bland–Altman plots of agreement for axial length (**a**), central corneal thickness (**b**), anterior chamber depth (**c**), lens thickness (**d**), mean keratometry (**e**), astigmatism (**f**), vector J_0_ (**g**), vector J_45_ (**h**), corneal diameter (**i**) between the ZW-30 and IOLMaster 700. The mean difference is indicated by a solid blue line, and the 95% limits of agreements (LoAs) are indicated by the dashed red lines

**Fig. 4:**
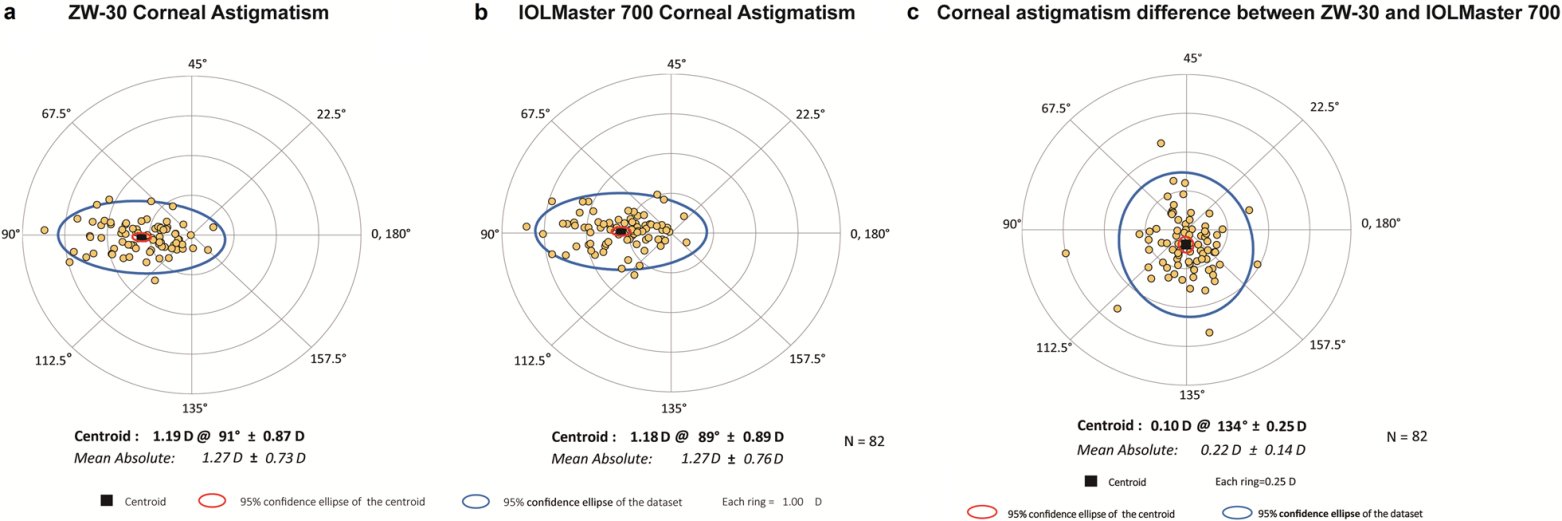
Double-angle plot of corneal astigmatism measured by the ZW-30 and IOLMaster 700. **a** Corneal astigmatism measured by ZW-30; **b** Corneal astigmatism measured by IOLMaster 700; **c** Corneal astigmatism measurement difference between the ZW-30 and IOLMaster 700

The comparison values and agreement data between the ZW-30 and IOLMaster 700 are reported in Table 3. Although there were statistically significant differences in AL, AQD, ACD, Km, J_45_ and CD values (*P* < 0.05), the 95% LoAs were relatively narrow (Fig. 3). The CCT, LT, AST and J_0_ values obtained by the two biometers were all similar with a maximum absolute 95% LoAs of 8.08 µm, 0.08 mm, 0.30 D, and 0.16 D, respectively (Fig. 3). The distribution of AST differences measured by the two devices are displayed in Fig. 4. The difference in the magnitude of AST was within a 0.50 D range for 95% of pairwise comparisons.

**Table 3:** Comparison between the ZW-30 and IOLMaster 700

Parameter	Mean ± SD	*P* value*	95% LoA✝
AL (mm)	0.00 ± 0.01	**0.014**	− 0.02 to 0.02
CCT (μm)	0.29 ± 3.97	0.506	− 7.49 to 8.08
AQD (mm)	− 0.01 ± 0.03	**0.000**	− 0.07 to 0.04
ACD (mm)	− 0.01 ± 0.03	**0.000**	− 0.07 to 0.04
LT (mm)	0.01 ± 0.04	0.136	− 0.07 to 0.08
Km (D)	0.07 ± 0.12	**0.000**	− 0.16 to 0.30
AST (D)	0.00 ± 0.15	0.795	− 0.30 to 0.29
J_0_ (D)	0.00 ± 0.08	0.887	− 0.16 to 0.16
J_45_ (D)	− 0.05 ± 0.09	**0.000**	− 0.23 to 0.13
CD (mm)	− 0.15 ± 0.12	**0.000**	− 0.39 to 0.10

*AL* = axial length; *CCT* = central corneal thickness; *AQD* = aqueous depth; *ACD* = anterior chamber depth; *LT* = lens thickness; *Km* = mean keratometry; *AST* = astigmatism; *CD* = corneal diameter; *SD* = standard deviation; *LoA* = limits of agreement. *Paired t-test. ✝Bland–Altman plot. Boldface values indicate statistical significance

## Discussion

The new dynamic real-time visualization optical biometer ZW-30, using SS-OCT technology to obtain eye measurements, may be a powerful tool for clinical application. The aim of this study was first to evaluate the repeatability and reproducibility of this biometer, then to evaluate its agreement with another commonly adopted device utilizing the same technology, the IOLMaster 700. Based on the results, we report two main findings: (1) the new biometer exhibited outstanding intraobserver repeatability and interobserver reproducibility (ICCs for almost all parameters were higher than 0.900); (2) all anterior parameters and AL measurement data were interchangeable between the new biometer and IOLMaster 700.

In our study, the AL values measured by the new SS-OCT based biometer showed the best repeatability and reproducibility among the available parameters with an ICC of 1.000. Although there was a statistically significant difference in AL measurement between the new device and the IOLMaster 700, the mean difference was rather small (0.00 ± 0.01 mm) and the maximum absolute 95% LoA was only 0.02 mm. This finding was in accordance with the study by Panthier et al. [10], who reported that the mean difference between the IOLMaster 700 and ANTERION (Heidelberg Engineering GmbH, Heidelberg, Germany) was 0.01 mm. In another study, Liao et al. [11] studied 103 healthy eyes and found that the mean difference between the IOLMaster 700 and OA-2000 was 0.00 ± 0.02 mm and the maximum absolute 95% LoA was 0.03 mm. Since a measurement error of 1 mm of AL value induces 2.5 D to 3.0 D deviation in IOL power calculation [10, 12], a 0.02 mm AL difference would reflect a 0.025 D to 0.030 D refractive error, which is difficult to distinguish for the human eye. Thus, the measuring of AL can be performed on the ZW-30 during clinical use. In addition, the scanning speed of ZW-30 is ten times faster than traditional scanning optical biological measurement instruments, requiring shorter patient cooperation time, and real-time data collection during the measurement process, resulting in better data correlation. The number of scanning lines and scanning range used for each measurement of multi-directional radiation scanning has doubled, resulting in a larger amount of data collection. For data with significant errors caused by blinking, eye rotation, and etc., the average value can be deleted to further ensure the accuracy of the data and analysis. Further, ZW-30 provides a real-time dynamic view of the entire eye from the cornea to the retina to determine whether the axial measurement is from the anterior surface of the cornea to the fovea of the retina, and thus reduces the risk of refractive error caused by incorrect measurement due to undetected poor fixation. Therefore, its utility in clinical practice is justified.

For CCT, our results found no significant difference between the ZW-30 and IOLMaster 700. The maximum absolute 95% LoA shown in the Bland–Altman plot was 8.08 µm. A study comparing the IOLMaster 700 with the Anterion obtained a maximum absolute 95% LoA of 19.05 µm [10]. Montes-Mico et al. [13], Liao et al. [11], and Cheng et al. [14], compared the IOLMaster 700 with the OA-2000 and found a maximum absolute 95% LoA of 19.72 µm, 24.67 µm, and 24.40 µm, respectively. Our result was much smaller than those reported in the above-mentioned studies. Considering the narrow 95% LoA and no significant mean difference values, we conclude that the ZW-30 and IOLMaster 700 can be used interchangeably for CCT measurements.

With regard to AQD, ACD and LT, the ZW-30 and IOLMaster 700 displayed excellent agreement, as the 95% LoA ranged, from − 0.07 mm to 0.04 mm, − 0.07 mm to 0.04 mm, and − 0.07 mm to 0.08 mm, respectively. ACD and LT are important parameters for calculating IOL power, especially with last generation formulas [12]. Besides, LT has been shown to play a role in ICL sizing [15]. A 1 mm error in ACD and LT measurement may lead to an approximately 1.0 D to 1.5 D difference of IOL power [16, 17], demonstrating that the differences revealed in the current study would not have any clinically detectable effect. The results are in good agreement with those reported by Omoto et al. [18], Liao et al. [11], and Dong et al. [19].

Despite the statistically significant difference (*P* < 0.001), good agreement between the ZW-30 and IOLMaster 700 was observed for Km (95% LoA: − 0.16 D to 0.30 D). Hua et al. [20] proposed that a 1.00 D measurement difference in Km would result in a 1.40 D difference of IOL power. Based on this, it could be inferred that a difference of 0.30 D in keratometric power would lead to an IOL power difference of approximately 0.42 D, which lies within the usual 0.50 D step increments of IOLs. Similar to our result, a previous study evaluated the Km values obtained by the IOLMaster 700 and OA-2000 and reported a mean difference of 0.00 ± 0.09 D with narrow LoA range [11]. Moreover, our team had compared the SS-OCT based device with the Scheimpflug based optical biometer (Pentacam AXL, OCULUS) and found comparable outcomes between both devices (95% LoA: − 0.48 D to 0.09 D) [21]. However, Tañá-Rivero et al. [22] analyzed the interchangeability between the IOLMaster 700 and the Pentacam AXL and demonstrated that the LoA range was wide and may have a significant impact, especially when selecting the Toric IOL power. Thus, the agreement between the ZW-30 and devices based on other corneal topography measurement principles in keratometric value measurement still needs to be further studied.

The mean difference values of AST, J_0_, and J_45_ measured in our study between the ZW-30 and IOLMaster 700 were 0.00 ± 0.15 D, 0.00 ± 0.08 D, and − 0.05 ± 0.09 D, among which the difference in J_45_ was statistically significant (*P* < 0.001). Nevertheless, the LoAs range were all narrow, with the widest being 0.29 D, marginally above the 0.25 D clinical limit. The double-angle plot also showed that the distribution of corneal AST measured by these two devices was similar, suggesting that the differences between these two devices can be considered clinically negligible.

Due to the popularity of phakic IOL implantation surgery, accurate measurements of CD have attracted the attention of surgeons [23]. In addition, an increasing number of new IOL formulas (such as Barrett Universal II and Holladay 2 formulas) also consider this parameter as one of the predicting variables [24]. Dong et al. [19] demonstrated a 0.24 ± 0.30 mm significant difference and a wide 95% LoA range from − 0.83 mm to 0.35 mm between the IOLMaster 700 and ANTERION. In another study, Shetty et al. [25] studied 127 eyes and found the maximum absolute 95% LoA to be 0.76 mm, indicating that the potential differences in CD value measurement should be non-negligible in clinical practice. Contrary to these studies, the current study concluded that the ZW-30 and IOLMaster 700 had high agreement in the measurement of the CD distances, with a narrow 95% LoA (− 0.39 mm to 0.10 mm). Therefore, we can conclude that the CD data are interchangeable and can be used for clinical practice.

There are several limitations in our study. The first drawback is that only normal unoperated myopic eyes were included. Hence, the conclusions could not be extended to those who had other types of refractive errors or had other ocular diseases history (such as keratoconus and cataract). Besides, the mean AL value in this study was 25.76 ± 1.10 mm (range: 23.31 mm to 29.16 mm). No short eyes (AL < 22.0 mm) and only three long eyes (AL > 28.0 mm) were included in the whole dataset, which warrant further studies. Finally, we only compared the new instrument with the same measurement technology based device (IOLMaster 700). More efforts will be made to compare it with other devices based on different technologies to better evaluate its precision.

## Conclusion

Our findings provide evidence of the high repeatability and reproducibility of the new device in measuring ocular parameters, as well as its excellent agreement with a similar SS-OCT based device, the IOLMaster 700. Future studies should include devices using other measurement principles and eyes under different conditions (i.e., keratoconus).

## Data Availability

The datasets used and/or analyzed during the current study are available from the corresponding authors on reasonable request.

## Abbreviations

IOL: Intraocular lens
SS-OCT: Swept-source optical coherence tomography
PCI: Partial coherence interferometry
OLCR: Optical low-coherence reflectometry
OLCI: Optical low-coherence interferometry
CDVA: Corrected distance visual acuity
AL: Axial length
CCT: Corneal central thickness
ACD: Anterior chamber depth
LT: Lens thickness
LED: Light-emitting diode
PD: Pupil diameter
CD: Corneal diameter
AQD: Aqueous depth
Kf: Flattest keratometry
Ks: Steepest keratometry
Km: Mean keratometry
AST: Astigmatism
SD: Standard deviation
Sw: Within-subject standard deviation
TRT: Test–retest variability
CoV: Coefficient of variation
ICC: Intraclass correlation coefficient
ASCRS: American Society of Cataract and Refractive Surgery
LoA: Limits of agreement
D: Diopter
